# Supplementary material for: Sarcopenia as a Prognostic Marker in Elderly Head and Neck Squamous Cell Carcinoma Patients Undergoing (Chemo-)Radiation
Source: Cancers (Basel). 2022 Nov 10;14(22):5536. doi: 10.3390/cancers14225536 (PMC9688610; doi:10.3390/cancers14225536)
Supplement: Supplementary file 1 [file cancers-14-05536-s001.zip › cancers-1984164-supplementary.pdf]

cancers MDPI

Supplementary Material

Sarcopenia as a Prognostic Marker in Elderly Head and Neck Squamous Cell Carcinoma Patients Undergoing (Chemo-)Radiation

Erik Haehl [1,2,3], Luisa Alvino [1], Alexander Rühle [1,2], Jiadai Zou [1,2], Alexander Fabian [1,2], Anca-Ligia Grosu [1,2] and Nils H. Nicolay [1,2,4,*]

[1] Department of Radiation Oncology, University of Freiburg—Medical Center, Robert-Koch-Str. 3, 79106 Freiburg, Germany
[2] German Cancer Consortium (DKTK) Partner Site Freiburg, German Cancer Research Center (dkfz), Neuenheimer Feld 280, 69120 Heidelberg, Germany
[3] Department of Radiation Oncology, University Hospital, LMU Munich, 81377 Munich, Germany
[4] Department of Radiation Oncology, University of Leipzig Medical Center, Stephanstr. 9a, 04103 Leipzig, Germany
* Correspondence: nils.nicolay@medizin.uni-leipzig.de

Table S1. Patient characteristics of a cohort of 280 elderly HNSCC patients undergoing curative (chemo)radiotherapy at the university hospital Freiburg from 2010-2019. n, number of patients; Karnofsky, Karnofsky Performance Status; HPV, human papillomavirus; *p*, *p*-value; n/a, not available.

n = 280		n	%
**Age**	65-74 years	178	63.6
	≥ 75 years	102	36.4
	median 72 years		
**Karnofsky**	100-90 %	153	54.6
	80-70%	104	37.1
	60-50 %	22	7.9
	40 %	1	0.4
	median: 90%		
**T-stage**	T1	37	13.2
	T2	63	22.5
	T3	73	26.1
	T4	100	35.7
	n/a	7	2.5
**N-stage**	N0	106	37.9
	N1	42	15.0
	N2	119	42.5
	N3	13	4.6
**HPV**	+	48	17.1
	-	69	24.6
	na	163	58.2
**Grading**	G1	11	3.9
	G2	167	59.6

	G3	91	32.5
	G4	1	0.4
**Localisation**	nasopharynx	5	1,8
	oropharynx	94	33.6
	hypopharynx	34	12,1
	Oral cavity	69	24,6
	larynx	45	16,1
	multi-level tumors	15	5,4
	salivary glands	9	3.2
	other	9	3.2

Table S2. Comparison of treatment related toxicities following the CTCAE v.5 for postherapeutic sarcopenic and non-sarcopenic elderly HNSCC patients undergoing curative (chemo)radiotherapy. p values given for group comparison with Mann-Whitney U tests and Fisher's exact tests. pL3MA, posttherapeutic cross-sectional skeletal muscle area at the third lumbal vertebra; pL3MI, posttherapeutic skeletal muscle index at the third lumbal vertebra; n, number of patients; *p*, *p*-value; n/a, not available.

		**pL3MA**					**pL3MI**				
**n=90**		**non-sarcopenic** (n=26)		**sarcopenic** (n=64)			**non-sarcopenic** (n=31)		**sarcopenic** (n=59)		
**Chronic toxicity grade**	**1**	19	73.1%	42	65.6%	*p*=0.495	23	74.2%	38	64.4%	*p*=0.348
	**2**	20	73.9%	42	65.6%	*p*=0.297	22	71.0%	40	67.8%	*p*=0.759
	**3**	6	23.1%	16	25.0%	*p*=0.848	9	29.0%	13	22.0%	*p*=0.465
	**4**	0	0	0	0		0	0	0	0	
**Chronic toxicity grade 3/4**		6	23.1%	16	25.0%	*p*=0.539	9	29.0%	13	22.0%	*p*=0.314
**Chronic max. toxicity**	**0**	2	7.7%	9	14.1%	*p*=0.351	1	3.2%	10	16.9%	*p*=0.160
	**1**	1	3.8%	10	15.6%		4	12.9%	7	11.9%	
	**2**	17	65.4%	29	45.3%		17	54.8%	29	49.1%	
	**3**	6	23.1%	16	25.0%		9	29.0%	13	22.0%	
	**4**	0	0	0	0		0	0	0	0	
